# Calomel in Hepatic Diseases

**Published:** 1874-05

**Authors:** H. C. Wood


Calomel in Functional Hepatic Diseases.—*By Dr. H. C. Wood.*—Physicians are divided into two schools on this subject; one saying that calomel has no effect on the liver, the other saying that it has. Those of the latter opinion are certainly in the majority, and embrace in their number by far the greater bulk of the active practitioners. Those who deny the action of mercury on the liver found their opinion upon experiments on animals; but, though much of our advance in therapeutics has been derived from this source, it cannot overthrow well-observed clinical facts.

Bennett, Gamgee, and others of the so-called "Edinburgh Committee," performed the experiments by making biliary fistulæ and giving calomel and other drugs. They found that calomel in small doses had no effect; in doses large enough to cause depression and other symptoms the amount of bile was diminished. But these experiments are unfair, because under abnormal conditions; and this alone would invalidate them.

Rohrig, a celebrated German author, opened dogs along the linea alba, drew out and opened the gall-bladder, and observed the secretion, which was kept up for several hours. To dogs about to be opened he gave calomel, with the result of greatly increasing the flow of bile. But I think that all these experiments should be cast aside. The only evidence to be relied upon is clinical.

Any of you may take ten or twenty grains of calomel, and your stools will become green. Some say that this green color is due to the formation of a sulphuret of mercury. I am not aware, however, that this has ever been proven for a single stool; and the question is not, Does *any* green calomel-stool contain mercury? but, Do *all* such stools? The evidence is most positive that many of the green stools of mercurial purgation contain no mercury in any form. Thus, Simon analyzed such fæces, and found bilivirdin and the other normal constituents of the bile, but no mercury; and Golding Bird and Michea did the same, with much the same result. Now, to find mercury is a very simple chemical problem, and it is impossible that the metal should have been overlooked by such eminent chemists as those mentioned, especially by Simon, a prince among organic chemists.

By some it has been thought that mercury simply causes bilious stools by increasing peristaltic action and sweeping out of the small intestines the bile naturally there present. I cannot believe this: it is notorious that resinous purgatives—drastics, far more active than calomel—do not produce "bilious stools." This clinical observation of every day has been confirmed by the chemical researches of Michea, and is enough of itself to indicate that calomel increases the secretion of bile. But still more cogent reasons are manifold for believing in the hepatic action of the drug. It is not rare to find persons whose livers are especially sensitive to the action of mercury; and in these I have known a moderate use of the drug to cause not merely violent bilious diarrhœa, but also the vomiting of large quantities of nearly pure bile.

Moreover, the history of disease and of its treatment seems to me to prove indisputably the truth of the view I am advancing.

It is a well-known fact that in juandice from obstruction the stools are almost white; and it has been over and over again experimentally proven that if in animals the bile be shut off from the intestines the stools lose their color.

You will often hereafter meet with cases in which slate-colored stools are passed, either with diarrhœa or constipation. This want of color is no doubt due to a want of bile in the alimentary canal, to a torpidity or absence of functional activity in the liver. If you give calomel to such a patient, you will find that the passages become black, or even green; and this may occur without any increase in the diarrhœa.

Many cases of serous diarrhœa, which have been treated indefinitely with astringents, etc., will be promptly cured by the exhibition of calomel.

To illustrate these points, I bring before you to-day these two men. I wish you to distinctly understand that the example I am about to give, although a striking one, is by no means a rare one; but the results mirror those which have been witnessed hundreds of times, and which I myself have seen over and over again. The first patient entered the hospital four weeks ago; he then had ten or twelve liquid, colorless, slaty, painful stools daily; there was no vomiting. He was given at intervals opium, bismuth, acetate of lead, tannic acid, and nitrate of silver, and his diet was regulated *secundum artem.* Under this treatment he improved very slowly, and continued to have liquid stools and to be utterly devoid of appetite for nearly three weeks, when the color of his passages gradually changed, and he is now just fairly convalescent.

This second man was admitted at the same time, from the same locality, and with exactly the same symptoms. For ten days he had similar treatment, without decided improvement. The astringents were then stopped, and he was put on blue mass, gr. ix daily, in three doses. In two days the stools were firmer, much less frequent, and, what is more important, their color was changed; on the third day the man was well.

The symptoms in the two cases were exactly similar; the disease had originated under precisely similar circumstances and causes; both patients being paupers in the out-wards, doing the same work, or rather enjoying the same idleness, eating at the same table, and having similar constitutions and similar past histories; yet one required three weeks of treatment without a mercurial, and the other, after pursuing a similar course as its fellow for ten days under the same treatment, is arrested in twenty-four hours by calomel *without* astringents.

These cases, gentlemen, are very strong in their contrast, but, I repeat, are not solitary ones, but examples of what I have seen over and over again. Diarrhœas apparently trivial resist treatment until you give calomel. Sometimes the mercurial suffices; often, however, after the biliary secretion has been started, it is necessary to use for a day or two bismuth and acetate of lead, or other astringents. The practical bearing of these remarks is that you should always inspect the stools, and, if they indicate the absence of bile, calomel should be given unless especially contra-indicated.

You will find sometimes that slate-colored stools and digestive disturbances coexist with constipation; and under these circumstances a mild mercurial course is often of great benefit. Again, sometimes green stools in adults replace the slate-colored passages of the diarrhœa of which we have been speaking. In such cases there is often excessive pain, especially if the diarrhœa has been stopped. Under these circumstances I have never found anything but a mercurial to act efficiently.

